# Land use and land cover changes influence the land surface temperature and vegetation in Penang Island, Peninsular Malaysia

**DOI:** 10.1038/s41598-022-25560-0

**Published:** 2022-12-08

**Authors:** Gbenga F. Akomolafe, Rusly Rosazlina

**Affiliations:** 1 School of Biological Sciences, Universiti Sains Malaysia, 11800 Penang, Malaysia; 2 Department of Plant Science and Biotechnology, Federal University of Lafia, Lafia, Nigeria

**Keywords:** Ecology, Environmental sciences, Space physics

## Abstract

The ecological changes in vegetation and land of an area can be monitored and managed through the assessment of its past and present land use and land cover (LULC). In this study, we assessed the changes in the LULC of Penang Island between 2010 and 2021. We also determined the corresponding impacts on the land surface temperature (LST) and vegetation index in the form of normalized difference vegetation index (NDVI). Landsat-5 and Landsat-8 were selected for the study. The LULC types were classified using both supervised and unsupervised multivariate maximum likelihood techniques. The LULC change analysis revealed a considerable increase in the urbanized areas (45.71%), a slight increase in the forests (1.57%) and a sizeable reduction in the agricultural/herbaceous areas (− 33.49) of the city within the stipulated period. The urbanized areas were observed to have the highest LST in 2010 and 2021 (28.75–34.0 °C) followed by the bare land (29.76–29 °C). The increase in temperature could have been driven by the reduction in the greenness of the city coupled with the openness of vegetation cover. Similarly, strong positive correlations were observed between the LST and NDVI in the urbanized areas (R^2^ = 0.92), and bare lands (R^2^ = 0.86). We, therefore, hypothesize that urbanization is the main driver of the LULC changes on Penang Island.

## Introduction

In recent times, the assessments of land use and land cover (LULC) changes have been used in the monitoring and management of ecological changes in different parts of the world^1^. Land use refers to the different human activities on the land which result in changes in the vegetation structure, water bodies, soil, rocks and other natural resources of an area^2^. Having accurate knowledge of the LULC of a place enhances the proper management of the challenges associated with the land. Apart from this, knowledge of the past present and future LULC changes also enables an appropriate estimation of the socio-economic and environmental impacts of such changes^3^. These LULC changes are direct products of increased global human activities and urbanization^4,5^.

Human activities are more impactful on the vegetation cover of terrestrial ecosystems thereby leading to environmental changes at local, regional and global levels^6^. These environmental changes also include an increase in the surface temperature due to the transformation of vegetation covers to other land use forms such as bare surfaces, solid surfaces, and agricultural lands^7,8^. It has been predicted that the land surface temperature of most parts of the world, especially developing countries will increase geometrically due to the impacts of pollution and urbanization in the year 2050^9^. Among other factors, population increase and uncontrolled and improper management of changes in LULC of urban areas are contributors to global climate change which increased surface temperatures. Hence, the assessment of LULC changes in an area will enable the understanding of the degree and spatial extent of anthropogenic changes there^6^.

The land surface temperature (LST) has been described as very essential in the assessment of the earth's surface features including the LULC and others^10^. Many studies have proved the influence of LULC on the LST of different parts of the world using remote sensing techniques^6–8^. Satellite remote sensing and geographic information systems are viable tools used for investigating the intensity of human impacts on the ecosystems through the mapping of LULC changes and vegetation indices within a stipulated period^11^. Remote sensing makes it easier and more economical to access data on vegetation and LULC changes in an area at a specific time^12^. These spatial data can then be managed and analyzed accurately using GIS techniques^13^. Landsat sensors such as Landsat 5 Thematic Mapper (TM), Landsat 7 Enhanced Thematic Mapper (ETM) and Landsat 8 Operational Land Imager (OLI) have been used to assess LULC and vegetation indices across the world^14^.

Studying the LST of an area could supply useful information on the human survival of such an area^15^. It could also provide information on the survival of crops because extreme climatic conditions negatively influence the growth, survival and productivity of crops^16^. Landsat data such as Landsat 8 has been very instrumental to the study of LST at both local and regional scales^17^. On the other hand, the normalized difference vegetation index (NDVI) has been used to measure the presence and dynamics of vegetation such as the green leaf area index, vegetation cover, green biomass and vegetation productivity^18^. It indicates the vegetation condition and predicts the productivity of plants in several areas of the world^19^. The NDVI works on the principle of electromagnetic radiation in which the greenness portion of the vegetation shows less reflectance in the visible spectrum because of the absorption of photosynthetic pigments. Consequently, it has a maximum reflectance in the near-infrared region^20^. In this study, the impact of LULC changes on the LST and vegetation of Penang Island between the years 2010 and 2021 was examined.

Penang Island was chosen considering that it houses the UNESCO world heritage centre (Georgetown). Georgetown has a rich and diverse cultural heritage; hence it was enlisted as a world heritage centre in 2008^21^. Over the years, this Island has witnessed lots of developmental transformations due to rapid industrialization and other anthropogenic influences^22^. It has been said that studying past events in the climate and land use change of an area will enhance proper deductions of the effects of such factors in the future^23^. Consequently, it became highly imperative for an assessment of the impacts of these anthropogenic changes on the vegetation of the famous city. Hence, the specific objectives include assessing the LULC changes in Penang over the 11 years; assessing the changes in LST and NDVI and; assessing the relationship between the LST and NDVI with regard to the LULC classes.

## Results

The overall accuracy and Kappa coefficient for the year 2010 are 85.76% and 0.85 respectively while those for the year 2021 include 88.53% and 0.89 respectively (Table 1).

**Table 1:** Accuracy assessment of LULC classification of Penang Island.

LULC class	2010		2021	
	Producer’s accuracy (%)	User’s accuracy (%)	Producer’s accuracy (%)	User’s accuracy (%)
Urbanized areas	78.51	80.45	90.13	88.23
Forests	87.32	85.42	89.55	87.21
Agricultural lands	77.56	85.81	80.77	87.54
Bare lands	70.16	82.13	75.21	86.72
Rocks	88.21	86.43	90.81	88.42
Water bodies	77.22	73.15	80.24	79.03
Overall accuracy	85.76%		88.53%	
Kappa coefficient	0.85		0.89	

**Figure 1:**
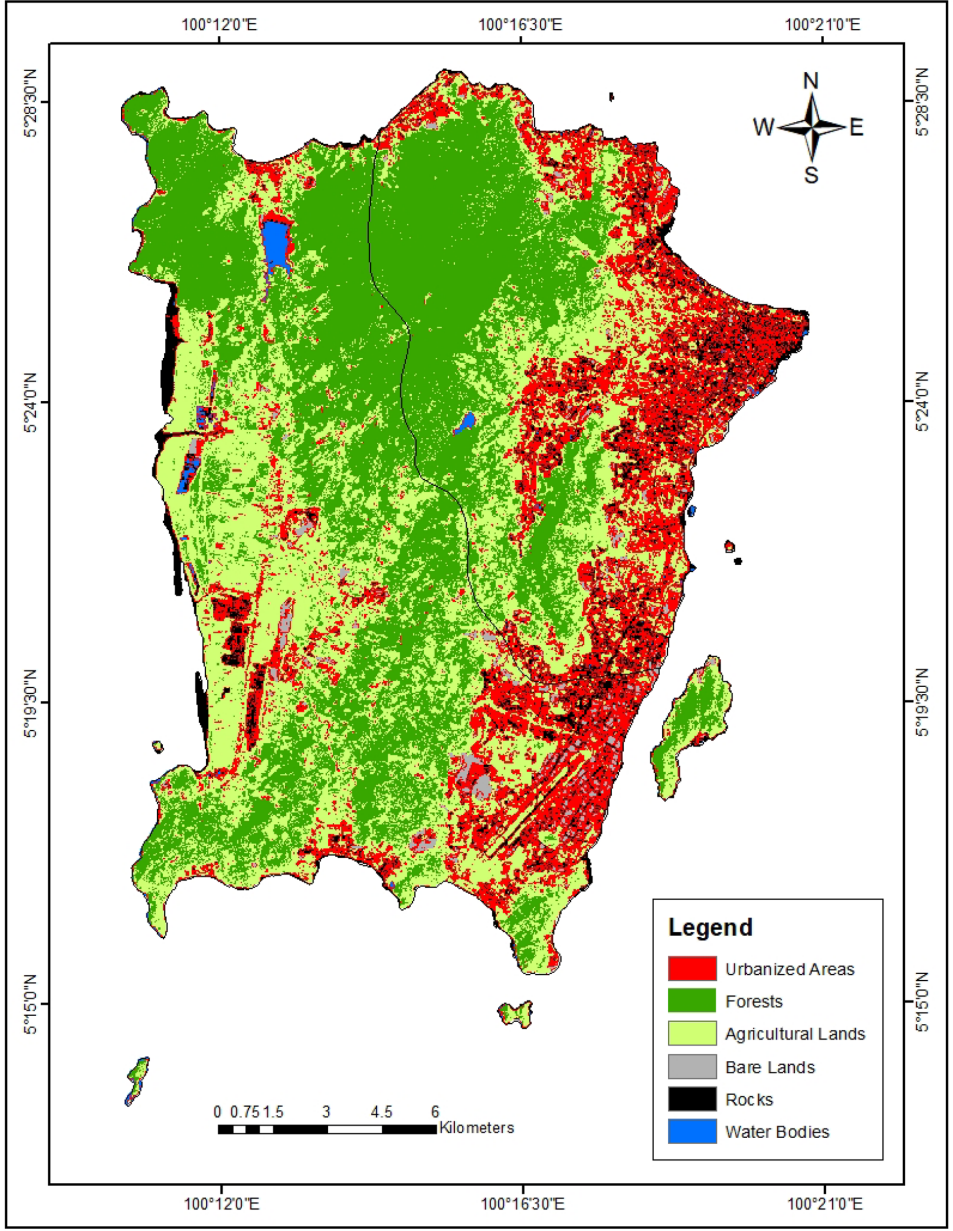
The LULC map of Penang Island in 2010 (created by the authors using ArcMap 10.8 software).

**Figure 2:**
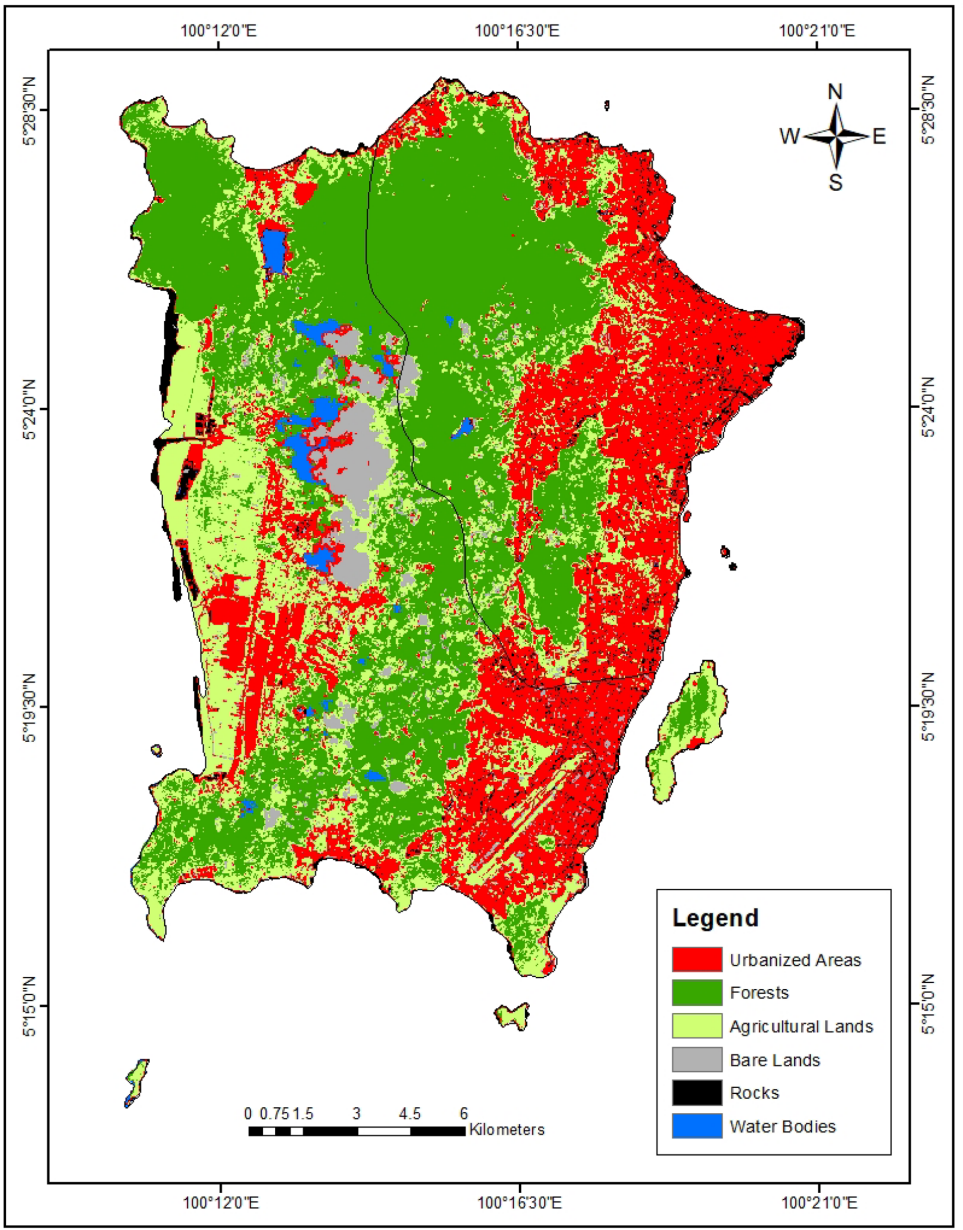
The LULC map of Penang Island in 2021 (created by the authors using ArcMap 10.8 software).

Regarding the producer’s accuracy (2010 and 2021), all the LULC classes were greater than 70%. Similarly, the user’s accuracies for all the LULC classes also exceeded 70%. This indicates that the classification of the LULC classes was achieved with very high accuracy. The results of the LULC classification of Penang Island (Table 2) indicated that forests have the highest area of 12025.44 ha (39.17%) in 2010 (Fig. 1) and 12213.70 ha (39.78%) in the year 2021 (Fig. 2) whereas the water bodies were found to have the lowest area of land (189.12 ha in 2010 and 385.27 ha in 2021).

**Table 2:** LULC area of Penang Island (2010–2021).

LULC class	2010		2021	
	Area (ha)	Area (%)	Area (ha)	Area (%)
Urbanized area	6111.62	19.90	8905.43	29.01
Forests	12,025.44	39.17	12,213.70	39.78
Agricultural lands	10,456.32	34.06	6954.51	22.65
Bare lands	425.44	1.39	1521.21	4.95
Rocks	1495.07	4.87	722.89	2.35
Water bodies	189.12	0.62	385.27	1.25
Total area	30,703.01	100	30,703.01	100

The analysis of the rate of change reveals a drastic increase in the urbanized areas which had a 45.71% increase over the 11 years of observation (Table 3). During this period, the urbanized area increased from 6111.62 to 8905.43 ha. This amounts to an annual increase rate of 4.16%. Also, the forest lands experienced a slight increase of 1.57% during this period. The other LULC classes with increasing land areas include bare lands (257.56%) and water bodies (12.93%). On the contrary, the agricultural land had a decrease of − 33.49% while the rocks had a decrease of − 51.65%.

**Table 3:** The annual rate of change in the LULC of Penang Island (2010–2021).

LULC class	Change in area (ha)	% Change	Annual rate of change (%)
Urbanized area	2793.81	45.71	4.16
Forests	188.26	1.57	0.14
Agricultural lands	−3501.81	−33.49	−3.04
Bare lands	1095.77	257.56	23.41
Rocks	−772.18	−51.65	−4.69
Water Bodies	24.46	12.93	1.18

cloud covers (the main challenge of remote sensing of tropical countries)^40^.

### LULC impact on LST

The satellite images studied have been characterized for the land surface temperature regarding the LULC classes. The average LST values as influenced by the LULC are presented in Table 4.

**Table 4:** Average LST (°C) and NDVI for different LULC classes.

LULC class	LST 2010	LST 2021	NDVI 2010	NDVI 2021
Urbanized areas	28.75	34.00	−0.43	0.10
Forests	23.60	31.40	0.53	0.42
Agricultural lands	24.63	31.90	0.36	0.43
Bare lands	29.76	29.00	−0.75	0.05
Rocks	25.86	32.50	−0.98	0.04
Water bodies	23.87	31.500	−0.97	−0.003

**Figure 3:**
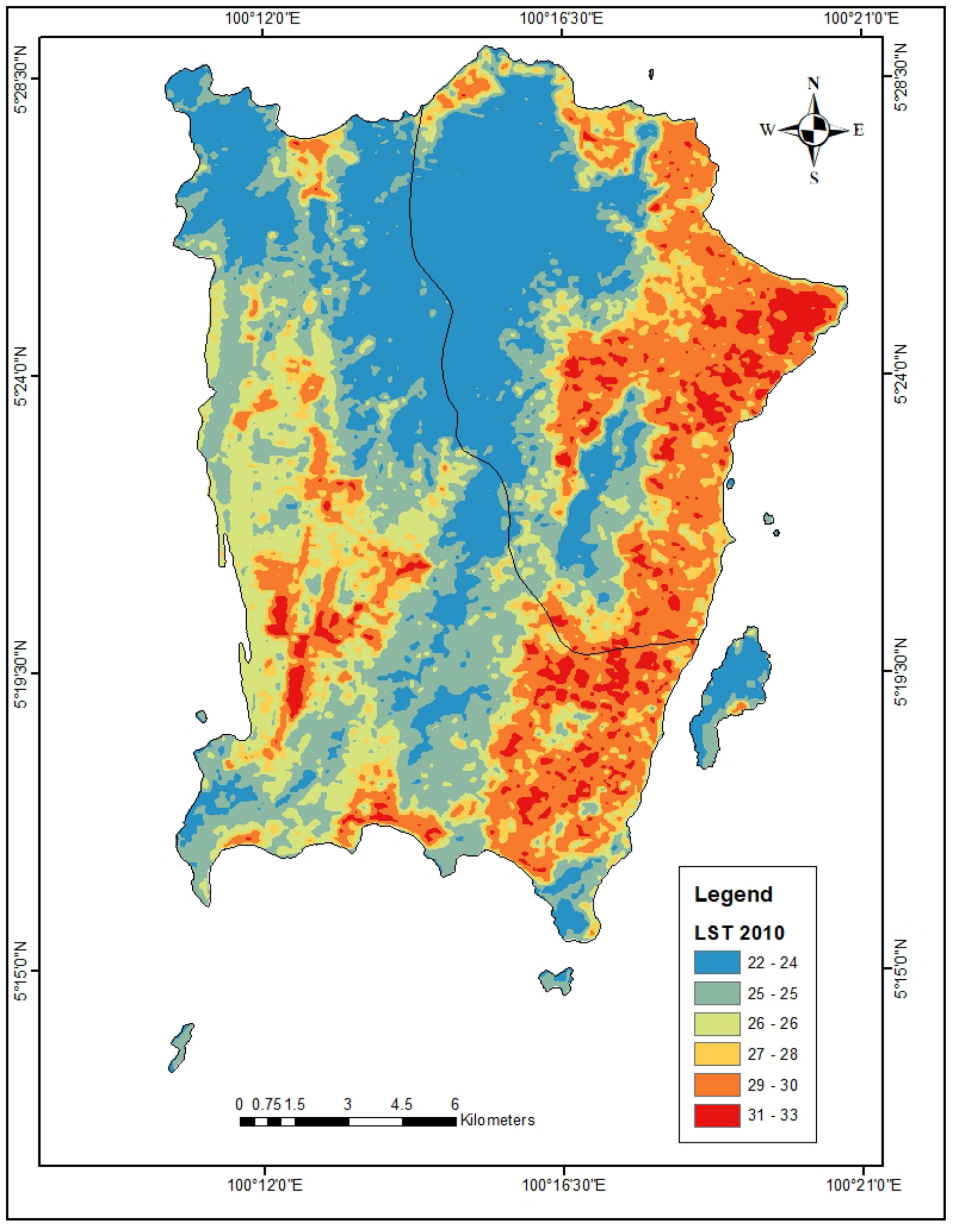
LST of Penang Island in 2010 (created by the authors using ArcMap 10.8 software).

**Figure 4:**
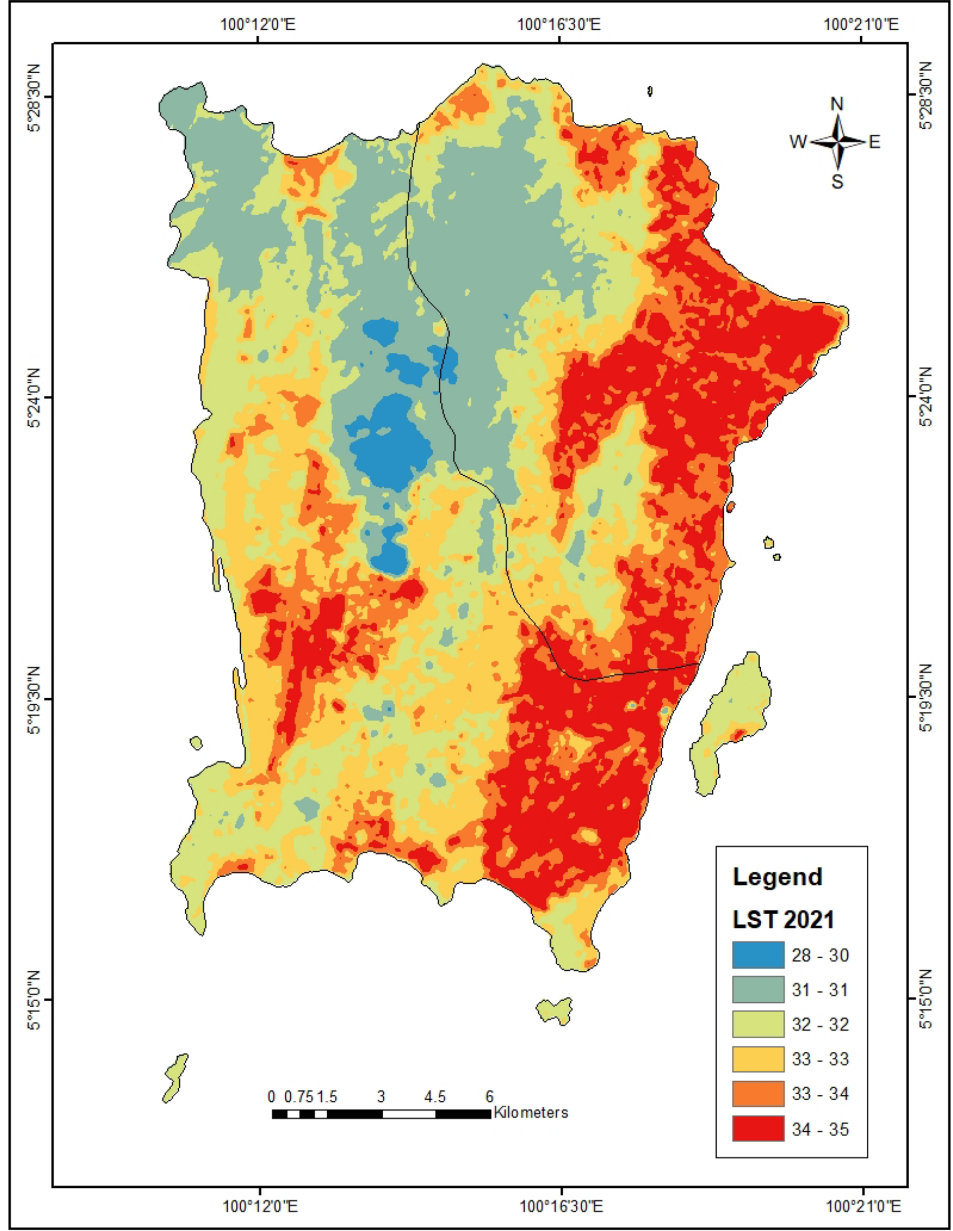
LST of Penang Island in 2021 (created by the authors using ArcMap 10.8 software).

**Figure 5:**
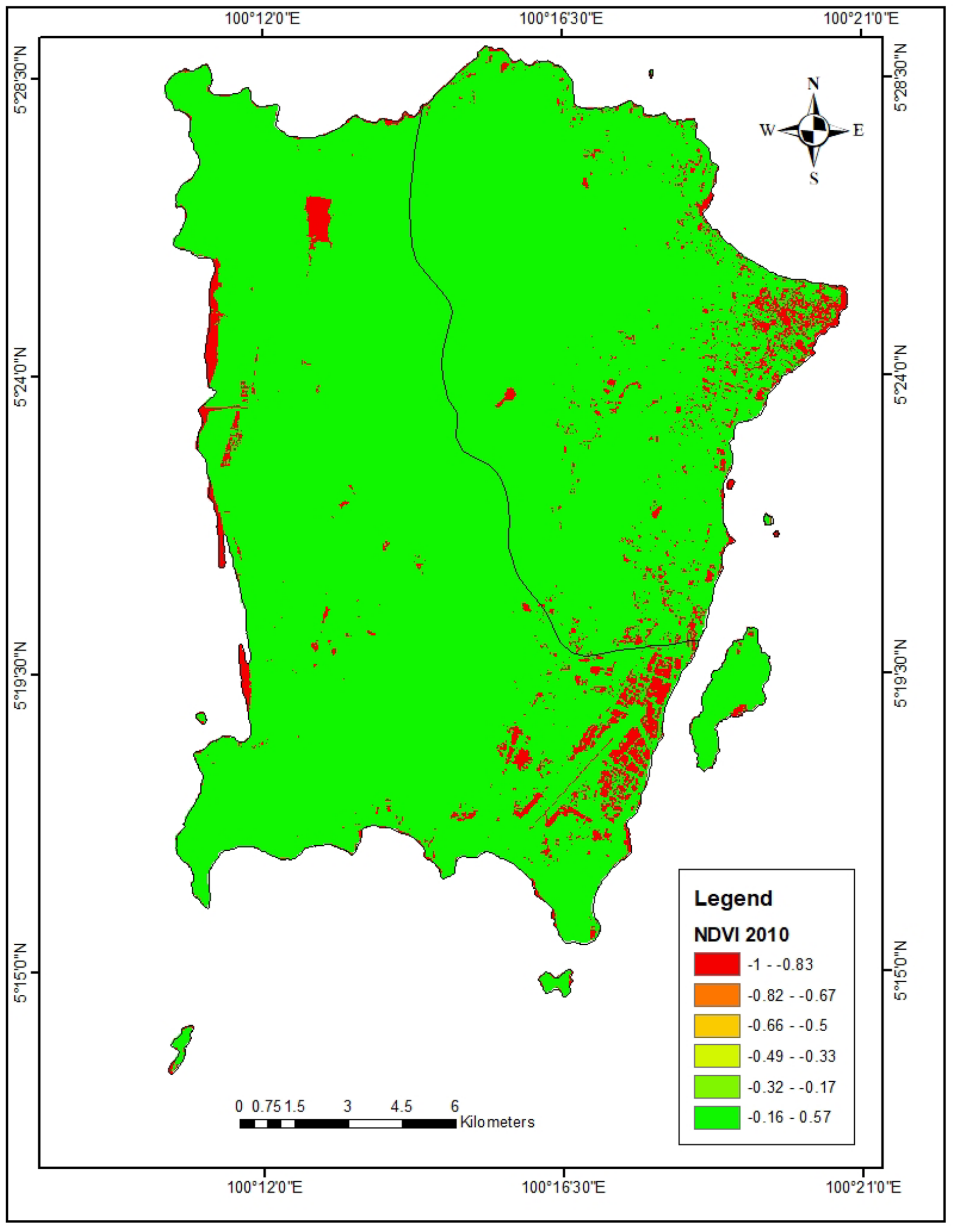
NDVI of Penang Island in 2010 (created by the authors using ArcMap 10.8 software).

**Figure 6:**
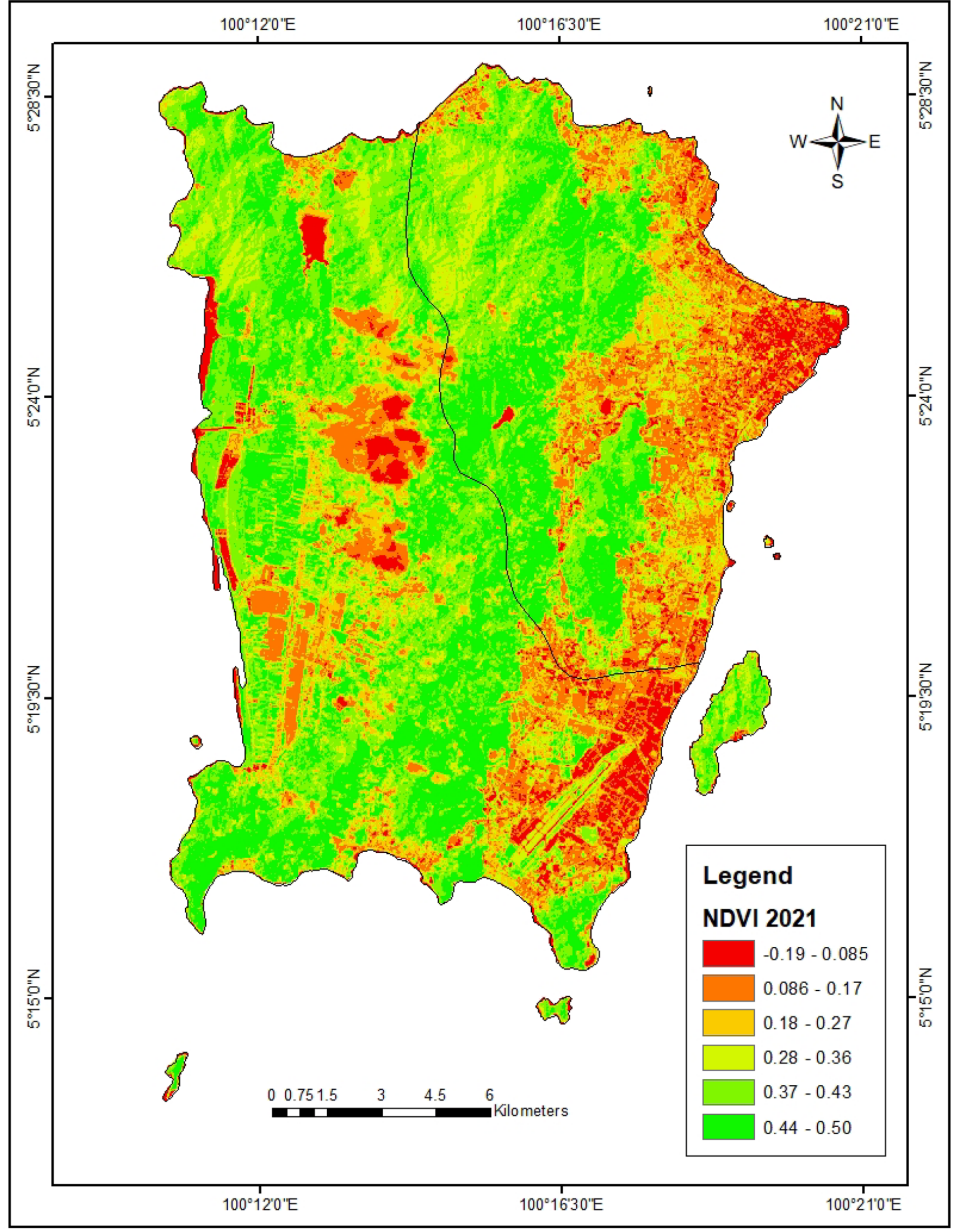
NDVI of Penang Island in 2021 (created by the authors using ArcMap 10.8 software).

In the year 2010, the bare lands and urbanized areas have the highest LST of 29.76 °C and 28.75 °C respectively (Fig. 3). Also in 2021, the urbanized areas exhibit the highest LST of 34.0 °C (Fig. 4). The forest lands have the lowest LST (23.60 °C) in 2010 which increased to 31.4 °C in 2021. Furthermore, the forest and agricultural lands were found to have the highest NDVI of 0.53 and 0.36 respectively in 2010 (Table 4, Fig. 5). This same trend was observed in the year 2021 where forest and agricultural lands had the highest NDVI (Fig. 6). Besides, the same LULC classes with lower NDVI values in 2010 still had lower NDVI in 2021.

### Relationship between LST and NDVI across LULC types

The relationship between the LST and NDVI is shown in Table 5. As revealed by the linear regression analysis, the LST had negative correlations with the NDVI values of forests, agricultural lands and bare lands in the year 2010.

**Table 5:** The relationship between LST and NDVI.

LULC Class	2010	2021
Urbanized areas	LST = 5.09NDVI + 29.19 (R^2^ = 0.53)	LST = 4.88NDVI + 34.39 (R^2^ = 0.92)
Forests	LST = −2.96NDVI + 23.47 (R^2^ = 0.14)	LST = −9.29NDVI + 27.5 (R^2^ = 0.20)
Agricultural lands	LST = −4.41NDVI + 24.44 (R^2^ = 0.32)	LST = −8.23NDVI + 35.37 (R^2^ = 0.24)
Bare lands	LST = −1.4NDVI + 29.55 (R^2^ = 0.04)	LST = 22.2NDVI + 27.69 (R^2^ = 0.86)
Rocks	LST = 2.06NDVI + 27.89 (R^2^ = 0.03)	LST = 0.91NDVI + 32.0 (R^2^ = 0.03)
Water bodies	LST = 11.73NDVI + 35.32 (R^2^ = 0.91)	LST = −1.09NDVI + 31.42 (R^2^ = 0.06)

In 2010, water bodies have the highest positive correlation between LST and NDVI (R^2^ = 0.91), followed by the urbanized areas (R^2^ = 0.53). The strongest negative correlation between LST and NDVI was observed in the agricultural lands (R^2^ = 0.32) and followed by the forest lands (R^2^ = 0.14). Interestingly, the forests and agricultural lands were discovered to have the lowest LST (23.6 °C and 24.63 °C respectively) and the highest NDVI values (0.21 each) in 2010. Also, a similar trend was observed in 2021 where LST exhibited strong negative correlations with NDVI in agricultural lands (R^2^ = 0.24) and forests (R^2^ = 0.20). The strongest positive correlation between the LST and NDVI was observed in the urbanized areas (R^2^ = 0.92), followed by the bare lands (R^2^ = 0.86).

## Discussion

The use of a remote sensing approach in the assessment of the impacts of LULC changes on the LST and vegetation cover of an area is beneficial in enhancing appropriate land management decisions^9^. In this study, the agricultural (herbaceous) land and rocks experienced a drastic decrease in the land areas during these 11 years. The loss of agricultural lands and rocks could have been gained by the urbanized areas. A similar study revealed that Penang has a track record of increased urbanization due to the vision of the State Government to make the State a renowned World Trade Centre^24^. This has led to a great influx of investors into the State, hence leading to the industrialization of the State. This Penang Island has already been described as the most developed part of the State comprising the international airport, factories, and many residential buildings^22^. Another study on land use and land cover changes in the capital city of Malaysia (Kuala Lumpur) revealed the same trend of rapid loss of green areas to urbanized areas over the last 15 years^9^. This high rate of urbanization did not only affect the vegetation cover of the city but also increased the pollution impacts on the city.

Landsat data are known to be very useful in understanding the impacts of LULC on the LST of an area^9^. The increase in the LST of this study area is similar to the previous work which showed that the urbanized areas of Penang Island have the highest LST in 1999 and 2007^24^. A similar observation was recorded in other cities in which the urban areas had the highest LST^10,25^. This means that the change in the urbanization and bare lands of the Island has influenced the LST. This is also caused by the loss in the vegetation cover of the agricultural or herbaceous lands to materials such as concretes, stones and tars used for urbanization. The lower LST values exhibited by forests and agricultural lands are attributed to their contributions to the photosynthetic pool of the area, thereby reducing the heat^24^. Therefore, urbanization involving buildings incorporated with vegetation (green buildings) and less concrete structures has been suggested as a way of reducing the LST of an area^26,27^.

It has been reported that the strength of the correlation is revealed by the linear regression coefficient^28^. The negative correlations observed between the LST and NDVI at the LULC classes indicate that the higher the surface temperature, the lower the vegetation cover or biomass of those LULC types^24^ and vice versa. Areas with high NDVI have been described as having enough vegetation cover which produces cooling effects thereby reducing the surface temperature^22^.

## Conclusion

From this study, it has been revealed that Penang Island had a considerable increase in the urbanized areas and bare lands coupled with a greater loss in the green areas (particularly the agricultural/herbaceous lands). The forests in this city only had a slight increase in land area. This is somehow commendable as the city was able to maintain its forest lands despite the rapid urbanization. However, the loss of agricultural or herbaceous lands to urbanization is also worrisome. This is because the agricultural/herbaceous lands also had roles to play in ensuring the maintenance of the vegetation cover / photosynthetic productivity of the city. Also, urbanization can be inferred as the main driver of the changes in the LULC of Penang Island. Due to the importance of urbanization, there is a need for an appropriate policy in managing land use to ensure balance in the transformation of LULC classes. This study hereby recommends that policymakers should ensure that the greenness of the city is maintained through the regulation of urban development.

## Methods

### Study area

**Figure 7:**
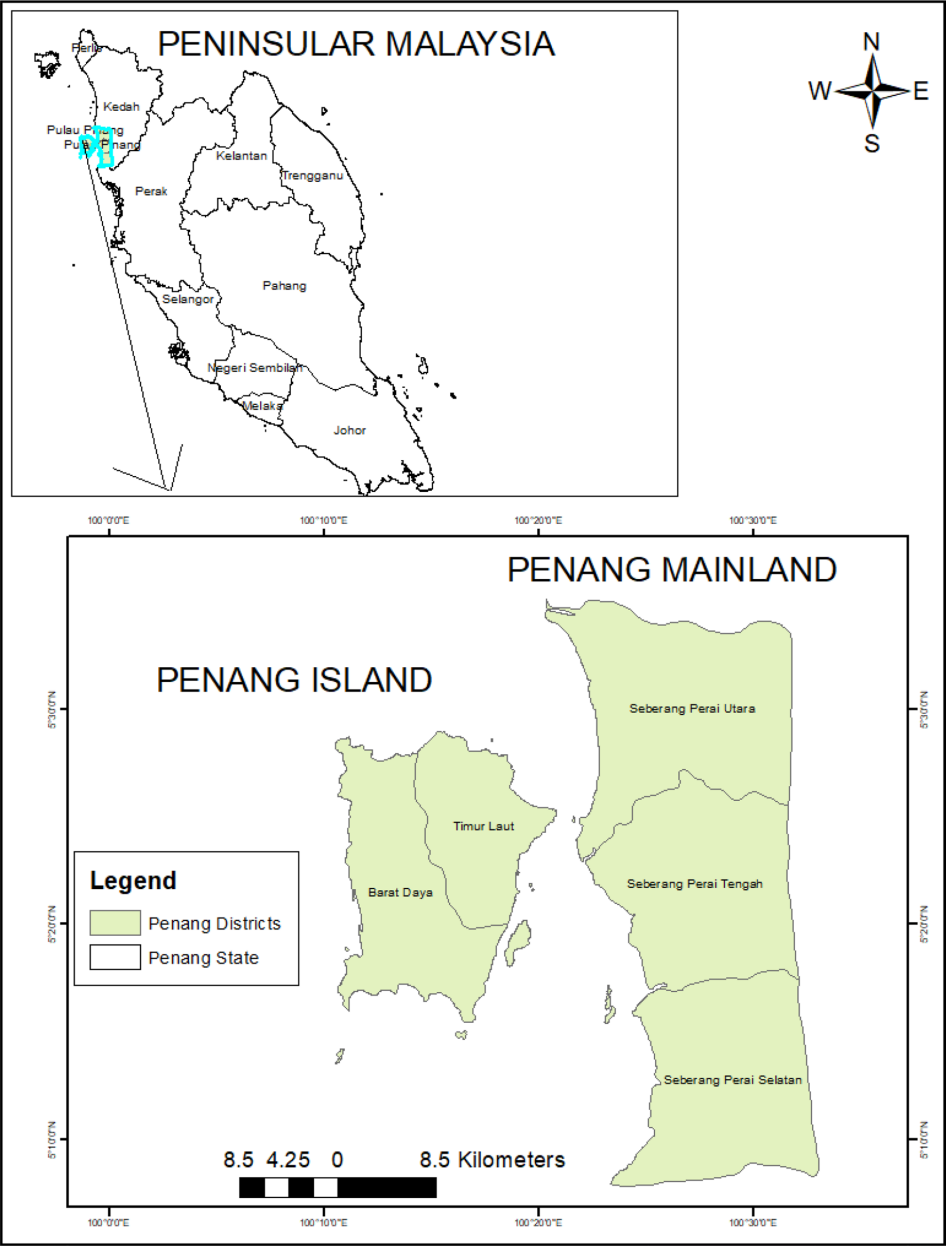
The map of Penang State showing the Penang Island (created by the authors using ArcMap 10.8 software).

Penang is situated in the northern part of Peninsular Malaysia and lies within the latitudes 5°12'N to 5°30' N and longitudes 100°09'E to 100°26'E (Fig. 7). Penang with a land area of 295 Km^2^, has an estimated population of 720,000 and is regarded as the most populated island in Malaysia. Penang shares the same border on the north and east with Kedah State and the south with Perak State. There are two main parts of Penang State: Penang Island and the mainland which is also regarded as Seberang Perai. These two parts of the State are connected by the two bridges. The eastern part of Penang Island is the most urbanized area comprising industries, commercial centres and residential buildings. However, the western part is less developed comprising mainly hilly terrain and forests^22^. This study is focused on the Island part of Penang. This island is endowed with a yearly equatorial climate (hot and humid). It has a mean annual temperature ranging between 27 and 30 °C while the mean annual relative humidity ranges between 70 and 90%. Also, the mean annual rainfall is about 267–624 cm.

### Data acquisition

**Figure 8:**
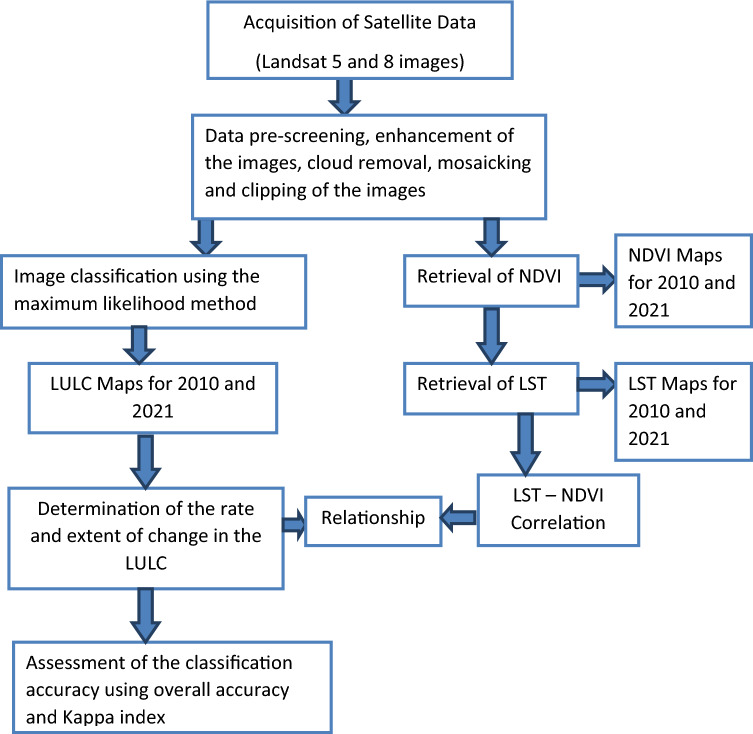
The flow chart of the methodology.

The flow chart of the methodology is presented in Fig. 8. Landsat satellite images were used for the assessment of changes in land use covering a period of 2010–2021 (11 years).

These images were gotten from the website of the United State Geological Survey (https://earthexplorer.usgs.gov). The Landsat images include the Landsat 5 TM (thematic mapper) and Landsat 8 OLI / TIRS (operational land imager / thermal infrared sensor). These were downloaded from the Landsat level 1 dataset (Table 6) with additional criteria which reduced the.

**Table 6:** The characteristics of the satellite data used.

Satellite	Path/Row	Sensor	Number of bands	Date captured by sensor	Spatial resolution
Landsat 8	128/56	OLI/TIRS	11	14th February 2021	30 m
Landsat 5	128/56	TM	7	16th February, 2010	30 m

### Determination of LST and NDVI for Landsat 5 and 8

Band 6 of Landsat 5 and band 10 of Landsat 8 were used for the determination of the land surface temperature (LST). The LST and normalized difference vegetation index were determined using the following steps:

#### Conversion of top of atmosphere (TOA) radiance

Using the radiance rescaling factor, thermal infra-red digital numbers were converted to TOA spectral radiance using the equation below^29^: $\frac{Red - NIR}{{Red + NIR}}$ $\frac{Red - NIR}{{Red + NIR}}$ For Landsat 8,

1 $$ {\text{L}}\lambda = \left( {{\text{ML}} \times {\text{ Qcal}}} \right) + \left( {{\text{AL}} - {\text{Oi}}} \right) $$

For Landsat 5,

2 $$ {\text{L}}\lambda = \left( {{ }\frac{{{\text{LMax}}\lambda - {\text{LMin}}\lambda }}{{{\text{QcalMax}} - {\text{QcalMin}}}}} \right) \times \left( {\left( {{\text{Qcal }} - {\text{QcalMin}}} \right) + {\text{LMin}}\lambda } \right) $$

where L_λ_ is TOA spectral radiance, ML is radiance multiplicative band Number, AL is radiance add band number, Q_cal_ is quantized and calibrated standard product pixel values (DN for band 6 or band 10), O_i_ is the correction value for the respective bands, LMax_λ_ is spectral radiance scaled to Q_cal_Max, LMin_λ_ is spectral radiance scaled to Q_cal_Min, Q_cal_Max is maximum quantized calibrated pixel value, and Q_cal_Min is minimum quantized calibrated pixel value.

#### Conversion to TOA brightness temperature (BT)

Spectral radiance data were converted to TOA brightness temperature using the thermal constant values in the Metadata file^29^.

Kelvin (K) to Celcius (°C) degrees

3 $$ BT = {\raise0.5ex\hbox{$\scriptstyle {K2}$} \kern-0.1em/\kern-0.15em \lower0.25ex\hbox{$\scriptstyle {\ln \left( {\frac{K1}{{{\text{L}}\lambda { } + { }1}}} \right)}$}} - 273.15 $$

where BT is the Top of atmosphere brightness temperature (°C), Lλ is TOA spectral radiance (W.m^−2^ .sr^−1^ .µm^−1^)), K1 is the K1 constant band number, and K2 is the K2 constant band number. For Landsat 5, K1 is 607.76, and K2 is 1260.56.

#### Normalized difference vegetation index (NDVI)

The Normalized Difference Vegetation Index (NDVI) is a standardized vegetation index which reveals the intensity of greenness and surface radiant temperature of the area^30,31^. The index value of NDVI usually ranges from − 1 to 1. The higher NDVI value indicates that the vegetation of the area is denser and healthier. This shows that the NDVI values of normal healthy vegetation range from 0.1– 0.75, while it is almost zero for rock and soil, and negative value for water bodies^24^. The NDVI is calculated using the followings:

4 $$ {\text{NDVI }} = \frac{{\left( {{\text{NIR }}{-}{\text{ RED}}} \right){ }}}{{\left( {{\text{NIR }} + {\text{ RED}}} \right)}} $$

In Landsat 4–7

$$ {\text{NDVI }} = \, \left( {{\text{Band 4 }}{-}{\text{ Band 3}}} \right) \, / \, \left( {{\text{Band 4 }} + {\text{ Band 3}}} \right) $$

In Landsat 8

$$ {\text{NDVI }} = \, \left( {{\text{Band 5 }}{-}{\text{ Band 4}}} \right) \, / \, \left( {{\text{Band 5 }} + {\text{ Band 4}}} \right) $$

where: RED = DN values from the RED band, and NIR = DN values from the Near Infra-red band.

#### Land Surface Emissivity (LSE)

Land Surface Emissivity is the average emissivity of an element on the surface of the earth calculated from NDVI values.

5 $$ {\text{PV }} = \left\{ {\frac{{\left( {{\text{NDVI }} - {\text{ NDVImin}}} \right)}}{{\left( {{\text{NDVImax }} - {\text{ NDVImin}}} \right)}}} \right\}^{2} $$

where PV is the Proportion of vegetation, NDVI is the DN value from the NDVI image, NDVI_min_ is the minimum DN value from the NDVI image, and NDVI_max_ is the maximum DN value from the NDVI image.

6 $$ {\text{E }} = \left( {0.004{ } \times {\text{PV}}} \right) + 0.986 $$

where E is land surface emissivity, PV is the Proportion of vegetation, 0.986 corresponds to a correction value of the equation.

#### Land Surface Temperature (LST)

Land Surface Temperature (LST) is the radiative temperature which is calculated using top of atmosphere brightness temperature, the wavelength of emitted radiance and land surface emissivity.

7 $$ {\text{LST}} = {\raise0.5ex\hbox{$\scriptstyle {BT}$} \kern-0.1em/\kern-0.15em \lower0.25ex\hbox{$\scriptstyle {\left( {1 + \left( {{\lambda  } \times { }\frac{{{\text{BT}}}}{{{\text{c}}2}}} \right) \times {\text{ln}}\left( {\text{E}} \right)} \right)}$}} $$

Here c2 is 14388. The value of λ for Landsat 5 (Band 6) is 11.5 µm and Landsat 8 (Band 10) is 10.8 µm.

Where BT is the top of atmosphere brightness temperature, λ is the wavelength of emitted radiance, and E is land surface emissivity.

c2 = h*c/s (1.4388*10^–2^ mK = 14388 mK), h is Planck’s constant (6.626*10^34^ Js), s is Boltzmann constant (1.38*10^23^ JK), c is velocity of light (2.998*10^8^ m/s).

### Determination of land use and land cover (LULC) of the study area

The Landsat images were pre-screened and subjected to clipping and classification^32^. The boundary shape file of Penang was used to clip out the area of study.

#### Image classification

The unsupervised method involving a random assignment of sample training points and supervised methods of satellite image classification was employed in this study for determining the LULC types. This mixture of image classification methods has been reported as vital in achieving a high accuracy level^33^. Bands 5, 4 and 3 were used to classify Landsat 8 while bands 4, 3 and 2 were used for classifying Landsat 5. We used the extraction by mask in the spatial analyst tool of ArcMap 10.2.1 software to extract the study area from the selected bands of the Landsat satellite images. A widely used supervised image classification method was adopted for classifying the Landsat bands in this study^32,34^. The principle of operation of this method involves the identification of known sample training points which are then used to classify other unknown points with related spectral signatures^35^. The three monochromatic satellite bands were combined to produce the false colour composite (FCC) using the data management tool^36^. This involves drawing polygons on the LULC type to select the training points. The LULC types adopted for this study include urbanized areas, agricultural land, rocks, forests, bare surfaces, and water bodies. These were modified LULC types from IPOC Good Practice Guidance^37^. To achieve this, a minimum of 40 sample points were selected randomly for each category of LULC type^36^. Having prior knowledge of the study area assisted in the selection of the training points^38^.

The multivariate maximum likelihood classification (MLC) technique was used for transforming the images. Other image transformation techniques have been used by researchers. These include the fuzzy set classifier, neural networks (NN) classifier, extraction and classification of homogenous objects (ECHO) classifier, per-field classifier, sub-pixel classifier, decision trees (DTs), support vector machines (SVMs), minimum distance classifier (MDC) and so-on^39^. The adoption of any of these techniques is dependent on the knowledge of the area of study, band selection, accessibility of data, the complexity of the landscape, the classification algorithm, and the proficiency of the analyst^39^. We preferred MLC to other techniques in this study due to its reported high level of accuracy in tropical regions^32,34^. Another reason for choosing MLC is that it is readily incorporated in many widely used GIS software packages. This MLC algorithm operates based on assigning pixels to the highest probability class and establishing the class ownership of such pixels. It is also regarded as a parametric classifier whose data follows almost a normal distribution^39^. We ensured the accuracy of this classifier by assigning a large number of training sample points using our prior knowledge of the study area.

#### Description of the LULC categories

The urbanized area is the developed part of the study area. This includes houses, roads, railways, and industries. This is also known to be a settlement in other literature^40^. Agricultural land is the part of the study area dominated by agricultural activities and herbaceous plants and grasses. Agricultural land is generally a product of deforestation^36^. Rocks are part of the study area comprising solid mineral materials (rocks). Bare land is the bare soil which is either made open by natural or human activities.

Forests are parts of the study area dominated by trees. They can be primary or secondary forests depending on the rate of disturbances. According to^41^, forest land is an area having more than 0.5 ha of flora comprising trees (height is above 5 m) with a canopy greater than 10%. The forests in Penang are generally both primary and secondary^42^. Water bodies are parts of the study area covered by water seasonally or permanently. These include seas, rivers, lakes, ponds, streams, or reservoirs^40^.

#### Determination of change in the LULC

The rate and extent of change in the LULC of Penang within the periods under consideration were determined following the formula below^43^:

8 $$ {\text{Changed area }}\left( {{\text{C}}_{{\text{a}}} } \right) \, = {\text{ T}}_{{\text{a}}} \left( {\text{year 2}} \right) \, {-}{\text{ T}}_{{\text{a}}} \left( {\text{year 1}} \right) $$

9 $$ {\text{Changed extent }}\left( {{\text{C}}_{{\text{e}}} } \right) \, = {\text{ C}}_{{\text{a}}} /{\text{ T}}_{{\text{a}}} \left( {\text{year 1}} \right) $$

10 $$ {\text{Percentage of change }} = {\text{ C}}_{{\text{e}}} {\text{x 1}}00 $$

where T_a_ means the total area.

### Determination of relationship between LST and NDVI

The values of LST and NDVI at 20 random points of each LULC class were used. The relationship between the LST and NDVI across all the LULC classes in each year was determined using the bivariate linear regression analysis. This was done in Paleontological Statistical (PAST) package 3.0.

### Classification accuracy assessment

The classification accuracy was assessed by taking ground truth coordinate data of the LULC of the study area using a geographical positioning system (GPS) device (Garmin Etrex 10). These data were compared with the LULC classified in this study^32^. Consequently, an error matrix was generated. This normally uses ground truth data to explain the accuracy of the classified LULC. The error matrix comprises the user’s accuracy, the producer’s accuracy, overall accuracy and the Kappa index^32^.

The producer’s accuracy (omission error) represents the probability of the correctly classified reference pixel and it is determined using this formula below:

11 $${\text{Producer's accuracy }}\left( \% \right) \, = { 1}00\% \, - {\text{ error of omission}} $$

Also, the user’s accuracy (commission error) represents the probability that the classified pixel matches the one on the ground^36^ and it is determined using the formula below:

12 $$ {\text{User's accuracy }}\left( \% \right) \, = { 1}00\% \, - {\text{ error of commission}} $$

The statistical accuracy of the matrix was determined using the Kappa coefficient^44^. This Kappa coefficient ranges from − 1 to + 1^45^. Therefore, the overall accuracy of the classification was determined by dividing the total number of correctly classified pixels by the total number of sampled ground truth data^40^.

## Data Availability

The datasets used and/or analysed during the current study available from the corresponding author on reasonable request.
