# Author Correction: Land use and land cover changes influence the land surface temperature and vegetation in Penang Island, Peninsular Malaysia

**DOI:** 10.1038/s41598-025-99225-z

**Published:** 2025-04-30

**Authors:** Gbenga F. Akomolafe, Rusly Rosazlina

**Affiliations:** 1 School of Biological Sciences, Universiti Sains Malaysia, 11800 Penang, Malaysia; 2 Department of Plant Science and Biotechnology, Federal University of Lafia, Lafia, Nigeria

Correction to: *Scientific Reports* 10.1038/s41598-022-25560-0, published online 08 December 2022

The original version of this Article contained errors.

References 15 and 16 were incorrectly given as

15. Vickers, N. J. Animal communication: When i’m calling you, will you answer too?. Curr. Biol. 27(14), R713–R715 (2017).

16. Romaguera, M. et al. Detecting geothermal anomalies and evaluating LST geothermal component by combining thermal remote sensing time series and land surface model data. Remote Sens. Environ. 204, 534–552 (2018).

The correct references are listed below:

15. Orimoloye, I. R., Mazinyo, S. P., Nel, W., & Kalumba, A. M. Spatiotemporal monitoring of land surface temperature and estimated radiation using remote sensing: human health implications for East London, South Africa. Environ. Earth Sci. 77, 77 (2018). 10.1007/s12665-018-7252-6.

16. Amin A. et al. Evaluation and analysis of temperature for historical (1996–2015) and projected (2030–2060) climates in Pakistan using SimCLIM climate model: Ensemble application. Atmos Res. 213:422–36 (2018). 10.1016/j.atmosres.2018.06.021.

The original Article has been corrected.

